# Deep Eutectic Solvents as Green Media for Catalyst Synthesis in Advanced Oxidation Processes

**DOI:** 10.3390/molecules31030421

**Published:** 2026-01-26

**Authors:** Bárbara Lomba-Fernández, Marta Pazos, Emilio Rosales, Ángeles Sanromán

**Affiliations:** CINTECX, BIOSUV Group, Department of Chemical Engineering, University of Vigo, Campus As Lagoas-Marcosende, 36310 Vigo, Spain; barbara.lomba.fernandez@uvigo.gal (B.L.-F.); mcurras@uvigo.es (M.P.); emiliorv@uvigo.es (E.R.)

**Keywords:** deep eutectic solvents, catalyst, advanced oxidation processes, water treatment, green solvents, chemical synthesis, sustainability

## Abstract

At present, the contamination of wastewater by persistent organic pollutants is a problem causing significant concern. Advanced oxidation processes have emerged as effective and innovative technologies for the degradation of these pollutants. In these processes, the synthesis and usage of an appropriate catalyst is essential to enhance the generation of reactive species and improve treatment efficiency. In this sense, the use of greener solvents in the synthesis procedure has attracted great interest in recent years, improving the catalyst performance and reducing the associated synthesis impact. Among them, deep eutectic solvents stand out for the synthesis of catalytic materials in advanced oxidation processes for water treatment, offering a sustainable alternative to traditional methods due to their unique properties and low environmental impact. This review summarizes recent advances in this field, highlighting primarily the methods for preparing new catalytic materials using deep eutectic solvents and their application in different types of advanced oxidation processes.

## 1. Introduction

Water is widely recognized as a natural, limited, and highly valuable resource. From the simplest microorganisms to the most complex ecosystems, all depend on this liquid for their survival, making it an indispensable and vital resource for sustaining human life, maintaining ecosystems, driving economic activities, and fostering social development [1,2,3]. For all these reasons, it should be preserved and managed responsibly to ensure that it can be used by present and future generations. However, the rapid population growth and the increase in industrialization observed in recent decades have exerted enormous pressure on water resources, leading to their scarcity, pollution, and degradation [4,5,6]. In this context, the implementation of sustainable water management strategies, particularly water reuse, has emerged as a key solution to mitigate water scarcity, reduce pressure on freshwater sources, and promote a more efficient and circular use of this essential resource.

**Figure 1:**
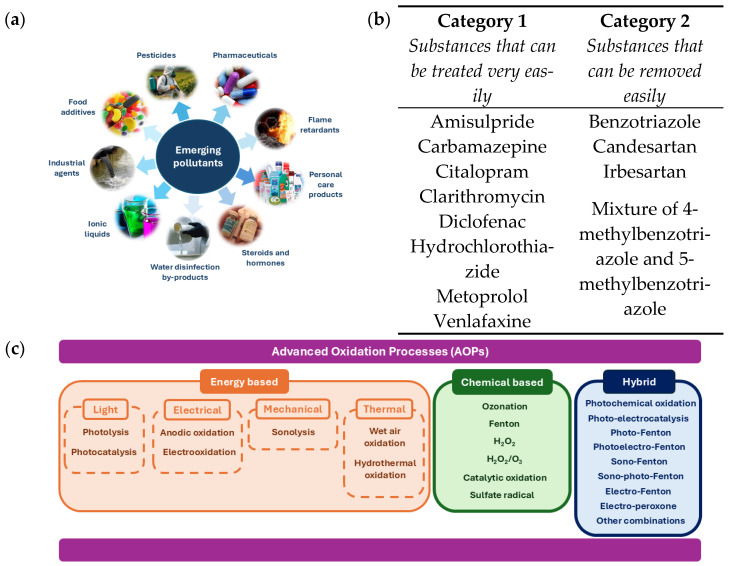
(**a**) Diagram of the main types of emerging pollutants, (**b**) compilation of microcontaminants designated for removal under 2024/3019 Directive [13], and (**c**) scheme of the essential types of AOPs.

Despite advances in wastewater treatment technologies, water reuse is increasingly challenged by the presence of persistent and poorly degradable pollutants in treated effluents. In recent years, the continuous discharge of waste into the environment has resulted in the accumulation of compounds that are not effectively removed by conventional wastewater treatment processes [7,8,9]. These pollutants, known as emerging pollutants, include various types of widely used products, as can be seen in Figure 1a, like medicines, pesticides, additives used in materials such as antioxidants or plasticizers, and domestic products such as detergents, cosmetics, fragrances, or creams. The presence of these emerging pollutants represents a serious threat to the safe reuse of treated wastewater, as they tend to accumulate in aquatic environments, causing ecosystem damage and potential risks to human health [10,11,12]. Consequently, there is an urgent need to develop and implement more effective treatment strategies to address the presence of emerging pollutants in wastewater and ensure the sustainability of water reuse practices.

This need has heightened concern, driving the search for solutions and the involvement of institutions. These have taken action by establishing new regulations, such as Directive (UE) 2024/3019 of the European Parliament and the Council of 27 November 2024, on urban wastewater treatment [13]. This new directive came into force at the beginning of 2025, and one of its novel aspects is the treatment requirements for the removal of micropollutants. To this end, it proposes their removal through quaternary treatment, which is mandatory for treatment plants that treat a load ≥150,000 p.e., as well as treatment plants that treat a load of more than 10,000 p.e. if there is a risk of accumulation of micropollutants in the aquatic environment, in accordance with a progressive implementation schedule with 100% of discharges subject to treatment by 31 December 2045. A minimum reduction of 80% is required for six substances that indicate the performance of quaternary treatment out of a total of 13 presented in Figure 1b. This proposal also recognizes the importance of monitoring the presence of per- and polyfluoroalkyl substances and microplastics in water, but does not require their elimination [13]. Its main objective is to strengthen treatment requirements and ensure a higher level of protection for the environment and human health.

This has prompted the search for efficient, economical, and environmentally friendly water treatment technologies to ensure compliance with regulations and high water quality. AOPs have emerged as powerful techniques for the degradation of persistent organic pollutants due to their high oxidation capacity, wide applicability, and fast reaction rates [14,15,16,17,18]. They are based on the generation of highly reactive oxidizing species, such as hydroxyl and sulfate radicals, which oxidize and degrade contaminants until partially mineralized, giving rise to intermediate products, or completely mineralized, yielding CO_2_, H_2_O, and inorganic compounds [15,19]. The versatility of these processes is due to the fact that there are different ways of producing these radicals (Figure 1c), and it is possible to choose which one to use, depending on the specific requirements for a given state of wastewater [17,20,21,22]. The effectiveness of AOPs is closely related to the design and performance of the catalysts, which are key determining factors. Often, they are synthesized using conventional solvent matrices that pose environmental and safety problems [16,23,24].

The development of economical, effective, and environmentally friendly solid-phase catalysts has become an important issue in chemistry, engineering, and materials science. Researchers around the world are actively working to create more environmentally friendly methods for catalyst synthesis. Their efforts focus on minimizing the use of hazardous solvents during synthesis and purification, avoiding toxic substances, reducing energy consumption, and promoting synthesis under ambient conditions [25]. Deep eutectic solvents (DESs) stand out in this context. They are liquid systems formed by the interaction of two or more components, typically a hydrogen bond donor and a hydrogen bond acceptor, in specific molar ratios to form a eutectic mixture with unique solvent properties. DESs represent a new class of environmentally friendly solvents with great potential to replace traditional catalysts and solvents [26,27,28]. For this reason, they have recently gained attention in the synthesis of catalytic materials, especially those applied in combination with AOPs for water treatment, offering a sustainable alternative to conventional methods.

**Figure 2:**
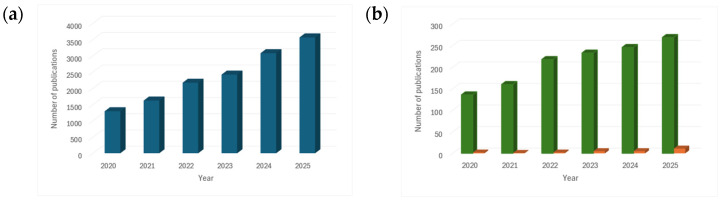
Evolution of the number of published articles in the last five years (2020–2025) related to keywords: (**a**) “deep eutectic solvents”, (**b**) “deep eutectic solvents” and “catalyst” (green bars), and “deep eutectic solvents” and “advanced oxidation processes” (orange bars).

In Figure 2a, the results of the literature search using Scopus^®^ and the keyword “deep eutectic solvents” for the period of 2020 to 2025 are presented. The steady increase in publications over the years reflects the growing interest in these materials in recent times. In Figure 2b, two sets of keywords were considered: “deep eutectic solvents” and “catalyst” (green bars), and “deep eutectic solvents” and “advanced oxidation processes” (orange bars). The first set clearly showed a significant increase in the number of publications, highlighting the progress and feasibility of DESs in the preparation of various catalysts. Although the growth in AOP-related publications is less pronounced, it is noteworthy that the number of publications in 2025 reached eleven, doubling the five reported in the previous year. This observation is particularly significant considering that this is still a very recent and relatively unexplored field. Therefore, in recent years, DESs have garnered considerable attention in the synthesis of catalytic materials and, more recently, for their application in AOPs for water treatment.

Therefore, the aim of this review is to explore the role of DESs in the synthesis of catalysts applied in AOPs, highlighting recent advances and future perspectives in terms of the preparation of new materials and their application in photocatalytic and electrochemical processes, thus providing an overview of the evolution of this field in recent years. To provide a comprehensive understanding, the review first introduces the fundamentals of DESs and their synthesis methods before discussing their applications in AOPs.

## 2. Fundamentals of DESs: Definition, History, Types, and Preparation Methods

**Figure 3:**
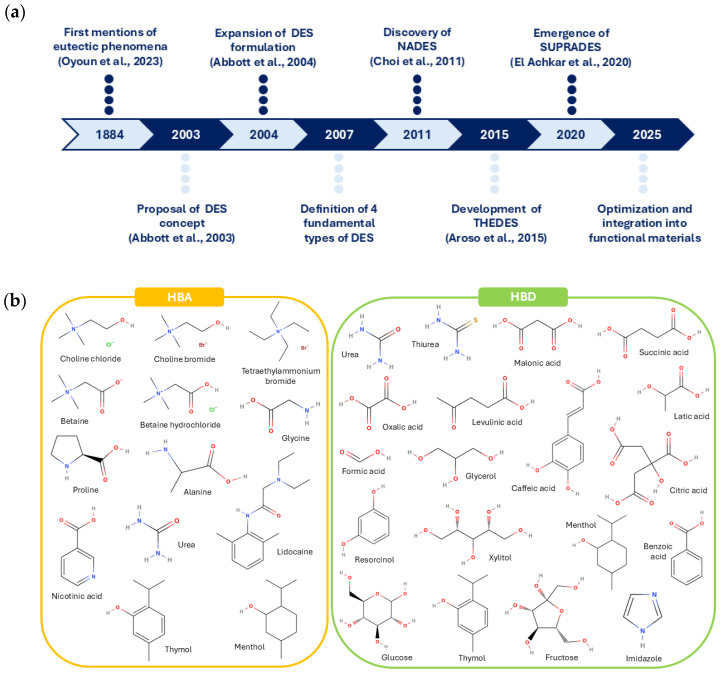
(**a**) Timeline of DESs from their origins to the present, highlighting their main discoveries. (**b**) HBAs and HBDs commonly used in the preparation of DESs [33,35,37,38,39,40].

DESs are binary or ternary mixtures formed through the interaction between a hydrogen bond acceptor (HBA) and a hydrogen bond donor (HBD). When combined in specific molar ratios, these components create a mixture of Lewis or Brønsted acids and bases, which consists of non-symmetric ions. These mixtures exhibit a lower eutectic point than that of an ideal liquid mixture, resulting in a reduced lattice energy. The depression of the melting point, compared to the individual components, is attributed to charge delocalization facilitated by hydrogen bonding. This unique characteristic enables DESs to remain in a liquid state at temperatures where the individual components would otherwise be solid [29,30,31]. The evolution of DESs from their origins to recent years is summarized in Figure 3a. The general expression to describe the formation of a DES is presented in Equation (1) [29,30,32].

Cat^+^ X^−^ · *z*Y (1)

where Cat^+^ represents a cation, X^−^ a Lewis base, Y a Lewis or Brønsted acid, and *z* indicates the number of Y molecules that interact with X^−^ to form complex anionic species.

As shown in Figure 3a, the earliest foundations date back to 1884, when Frederick Guthrie first introduced the term “eutectic” and defined it as a system composed of two or more components combined in such proportions that the resulting mixture exhibits a lower liquid temperature at a given composition than any other proportion [33]. In 2001, Abbott et al. [34] conducted pioneering research by heating several quaternary ammonium salts with zinc chloride and recording the freezing points of the resulting liquids. They found that choline chloride (ChCl) produced the lowest melting point (23–25 °C) when used as the ammonium salt [29]. Later, in 2003, the same authors formally introduced the term DES while studying mixtures of ChCl as the HBA and urea as the HBD. Their results showed that mixing ChCl (melting point 302 °C) and urea (melting point 133 °C) at a defined molar ratio of 1:2 produced a clear, deep eutectic liquid. They also observed that the melting points of these DES decreased to very low values, around 12 °C, significantly lower than the melting points of the individual components [35,36]. Following this discovery, additional DESs based on ChCl and carboxylic acids were characterized [37]. These studies sparked broad scientific interest, leading to substantial advances in the DES field. In 2011, Choi et al. [38] investigated the solubility of intracellular compounds that were insoluble in water and discovered natural DESs (NADESs). These solvents are composed of cellular metabolites, such as organic acids, alcohols, sugars, and amino acids. Later, in 2015, a therapeutic DES (THEDES) emerged when Aroso et al. [39] studied HBA and HBD mixtures incorporating active pharmaceutical ingredients with the aim of enhancing solubility and bioavailability. More recently, in 2020, supramolecular DESs (SUPRADESs) were developed [40]. This innovation arose from research aimed at expanding the properties of traditional DESs by incorporating supramolecular molecules, such as cyclodextrins, to improve the solubilization and stabilization of compounds.

Since the formal definition of the concept in 2003, a wide variety of HBAs and HBDs have been employed for the preparation of different DESs. Figure 3b summarizes the most commonly used components. Most HBAs are quaternary ammonium salts, such as choline chloride, choline bromide, choline nitrate, or choline acetate, while the majority of HBDs include sugars, amines, carboxylic acids, alcohols, or metal halides. Notably, the same molecule may act as either an HBA or an HBD depending on the second component present in the mixture; this is the case for compounds such as urea, menthol, and thymol [41,42].

### 2.1. Types

According to their composition, DESs can be classified into five types [36], as shown in Table 1. Type I DESs are composed of quaternary ammonium salts and metal chlorides. Type II are formed by mixing quaternary ammonium salts with hydrated metal chlorides. The most commonly used and studied DESs are Type III, due to their low toxicity and simple preparation. Generally, they are composed of ChCl as the HBA and various HBDs, such as carboxylic acids, amines, alcohols, sugars, and amides. Type IV combines features of Types II and III through the incorporation of hydrated metal halides and HBDs. Finally, more recently, hydrophobic DESs have been developed and classified as Type V. These consist of non-ionic HBAs, such as fatty acids, menthol, or thymol, and HBDs, such as long-chain alcohols and carboxylic acids. They are considered promising because they contain environmentally safe ingredients [29,31,36,42,43,44].

Meanwhile, several specialized categories of DESs have emerged. These include NADESs, composed of natural components; THEDESs, formed by pharmaceutical components such as ibuprofen, phenylacetic acid, or lidocaine; amino acid DESs (AADESs); polymeric DESs (PDESs), which contain plant-derived compounds, active pharmaceutical ingredients, amino acids, and polymers; and finally, SUPRADESs [33,45].

Advanced DES formulations represent a significant step forward in the development of sustainable solvents, as they allow for their properties to be tailored to specific industrial and environmental needs, thereby continuing the implementation of greener, safer, and more efficient chemical processes.

### 2.2. Methods of Preparation

DES preparation methods are characterized by their simplicity and cost-effectiveness, normally not requiring complex reaction mechanisms or subsequent purification involving the use of harmful or hazardous solvents [31,41,46]. DESs can be prepared in different ways; all of them have in common the mixing of two or more components in a specific stoichiometric ratio, usually without the need for solvents, and the addition of energy to the system for a certain period of time, either in the form of increased temperature, irradiation, or mechanical forces. In some cases, the initial components are dissolved in a solvent, usually water, and then heated under vacuum to evaporate the solvent or frozen and freeze-dried [45,47].

Up to now, numerous main approaches have been used to prepare DESs [29,33,48,49,50]. The conventional preparation method, known as heating and stirring, consists of heating and mixing solid HBA and HBD components until they melt, thereby creating the hydrogen-bond network required to form a homogeneous liquid phase [31,49,51]. The temperature needed can vary widely depending on the components and the procedure. This is the most commonly used, simple, and safe method [29,33]. Another method is grinding, which consists of mixing and crushing the components in a mortar until a clear liquid is formed [33,51]. Since the process is carried out at room temperature, it is ideal for heat-sensitive components [29,49]. The fastest preparation method is microwave-assisted synthesis, defined by adding stoichiometric amounts of HBA and HBD into a vial, which is then placed in a microwave for a specific time and at a given power [29,33,50]. There is also ultrasound-assisted preparation, which involves placing the components in a vial and applying ultrasound for a specific time and at a set temperature until a homogeneous liquid is obtained. After preparation, the DESs are kept at room temperature for 24 h to ensure the formation of a homogeneous mixture [29,33,50].

On the other hand, when using solvents, the freeze-drying preparation method stands out. It consists of dissolving the components in distilled water, freezing this solution at a very low temperature, and finally freeze-drying it [50,51,52]. This method is recommended for thermosensitive starting components and is not suitable for volatile reagents, since they are exposed to low pressures [33]. Finally, the vacuum-evaporation method is also widely used. It consists of dissolving the components in water, followed by removing the water through evaporation using a rotary evaporator and then drying it to a consistent weight in a desiccator [29,49,51,53]. Its main advantage is that lower temperatures are used than in the heating and stirring method, although complete water removal can be complicated and time-consuming [33].

Another existing but less commonly used method based on mechanical forces is twin-screw extrusion, which involves the use of a twin-screw extruder to continuously mix and grind the components [33]. This method is particularly advantageous due to the short exposure time to heat in the extruder, preventing the degradation of temperature-sensitive components, as well as its ability to operate continuously, which allows for easy scalability [33,50,54]. Crawford et al. [54] used this method to successfully produce a DES from ChCl and D-fructose.

Table 2 lists the advantages and disadvantages of each of these methods [29,31,50], as well as examples of research that have synthesized DESs using one of these procedures.

The time required to synthesize DESs varies between minutes and hours, depending on the preparation method and the initial components and their proportions. When selecting the preparation method to be used, temperature is a fundamental parameter and must be chosen carefully due to the possibility of degradation of the initial compounds. For example, this can be seen in the study by Crawford et al. [54], in which they compared two DESs made from the same components, ChCl and D-fructose, but using two different methods: heating and stirring, and twin-screw extrusion. They observed that DES obtained by the conventional heating and stirring method had a dark brown color, while the final DES obtained by twin-screw extrusion was transparent. This was because in the first case, the D-fructose degraded due to the temperature used.

Other previous studies have also shown that the preparation method can influence the physicochemical properties of DESs. In the study conducted by Florindo et al. [55], five DESs were prepared from ChCl (HBA) and different carboxylic acids (HBDs) using grinding, as well as heating and stirring methods. They concluded that DESs prepared by grinding were pure, while those obtained by the other method showed impurities in their structure. Therefore, selecting an appropriate preparation method is essential for obtaining homogeneous, pure, and stable DESs.

## 3. DESs in Advanced Oxidation Processes

**Figure 4:**
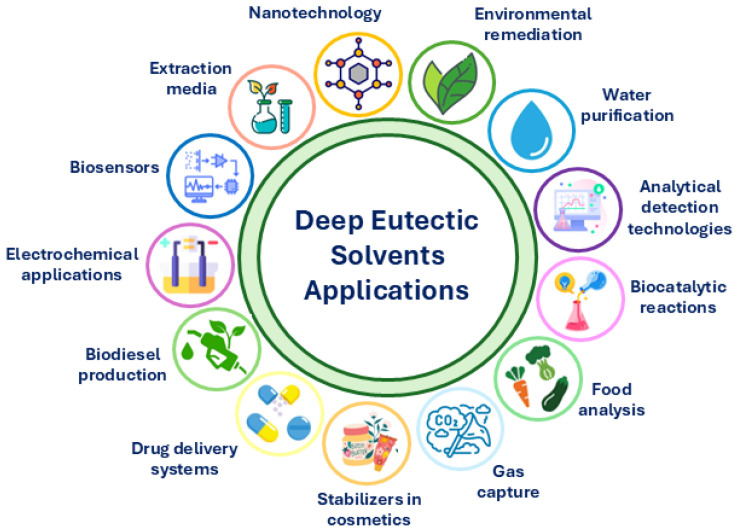
Key applications of DESs.

DESs have emerged as promising alternatives to conventional solvents owing to their low toxicity, biodegradability, and tunable physicochemical properties. By replacing volatile and toxic organic solvents, DESs help avoid the emission of volatile organic compounds and reduce the hazards associated with handling toxic substances, representing a significant step forward in environmentally sustainable chemistry. In addition, their reduced environmental impact and recyclability make them suitable for a broad range of applications across multiple fields, as can be seen in Figure 4 [31,42,67]. The potential applications of DESs are diverse, including their use in nanotechnology, CO_2_ capture [68,69,70], biodiesel production, as extraction media [71], in analytical detection technologies [72,73], electrochemistry, and water purification. More recently, DESs have been applied in the pharmaceutical industry, particularly in studies related to controlled drug release [74]. Significant progress has also been made in food analysis and sample preparation technologies [75], as well as in the synthesis of new catalytic and adsorbent materials [76,77].

Among these emerging applications, the use of DESs in the synthesis of catalysts has attracted particular attention due to their ability to influence the structural and chemical properties of the resulting materials. DESs have been successfully employed to produce metal nanoparticles, metal oxides, and supported catalysts with controlled size, morphology, and porosity. Acting not only as solvents but also as templating agents, stabilizers, or reducing agents, DESs enable the formation of highly dispersed and uniform catalytic materials. Their tunable composition further allows for the incorporation of heteroatoms or dopants, enhancing catalytic activity and selectivity. As a result, DESs have found applications in diverse catalytic processes, including hydrogenation, oxidation, electrocatalysis, and photocatalysis, demonstrating their broad potential in advanced material design and functional catalyst development [32,78,79,80].

While DESs have shown particular promise in the synthesis of catalysts for AOPs, their versatility extends to a wide range of catalytic applications, highlighting their potential beyond this specific area. In this context, DESs offer unique advantages for catalyst design, including improved control over morphology, composition, and surface properties, which are critical factors governing the efficiency of AOPs. Their ability to act as both solvents and templating agents allows for the synthesis of catalysts with highly uniform nanostructures and enhanced active site dispersion. However, despite these benefits, the practical application of DESs in catalyst preparation is not without challenges. Their high viscosity can hinder mass transport and mixing, while their hygroscopic nature and thermal sensitivity may affect the reproducibility and stability of the resulting materials. Moreover, complete removal or recycling of DESs after synthesis can be difficult, potentially impacting the purity and scalability of catalysts [80,81]. Accordingly, this and the following sections critically review and discuss the application of DESs in the synthesis and optimization of catalysts for AOP-based environmental remediation, highlighting both their potential and the limitations that must be addressed for practical implementation.

### 3.1. Role of DESs in Synthesis of Catalytic Materials for AOPs

Many conventional organic solvents used for catalyst synthesis are characterized by their toxicity and hazardous nature, being harmful to both human health and the environment. This has led, in recent years, to a growing search for more viable alternative solvents for the synthesis of materials with catalytic properties, with many of these studies focusing on DESs [80].

DESs exhibit a wide variety of unique properties that make them a promising material for carrying out catalyst synthesis. Some of these properties include high solubilization, viscosity, low volatility, high thermal stability, low toxicity, and density [82]. As previously explained, the HBA and HBD interact through hydrogen bonding to form a DES in specific molar ratios, meaning that the properties of DESs are primarily determined by the type of HBA and HBD used. By manipulating the size, type, purity, and molar ratio of the HBA and HBD, a large number of DESs with different properties can be prepared, which is of great interest because it makes DESs “designable” systems [82,83].

One of the characteristic properties of DESs is their low volatility, which makes them more stable and safer to handle compared to many conventional solvents. This is especially valued in catalysis, as it ensures the stability of the solvent during the reaction and helps improve the efficiency in catalyst synthesis. Furthermore, DESs are known for their reduced toxicity and for being more environmentally friendly than conventional solvents, as they are typically biodegradable [82,83,84].

The combination of these attributes, including their tunability, low volatility, low toxicity, and biodegradability, positions DESs as highly promising materials for the synthesis of novel and sustainable catalysts [80]. As summarized in Table 3, DESs can fulfill various functions in catalyst synthesis, acting as solvents, precursors, doping agents, or as structure- and morphology-directing components. The following sections provide a detailed analysis of these DES roles and examine their contributions to the design and development of catalysts for AOPs.

#### 3.1.1. DES as Solvent

One of the most remarkable properties of DESs is their ability to dissolve, disperse, and stabilize a wide variety of components, including transition metals and other reactive intermediates. This ability is attributed to their high polarity and hydrogen-bond-donating capacity, which allows them to form strong and stable complexes with metal ions and other catalytic species [80]. The high solubility of various precursors, such as Cu(NO_3_)_2_·H_2_O, NiCl_2_, CoCl_2_, FeCl_3_, or Zn(CH_3_COO)_2_·2H_2_O, makes them excellent solvents for hydrothermal synthesis [85,86]. Negi et al. [87] used a DES composed of urea, glucose, and water, and the NADES formed by urea, fructose, and water to solubilize Cu(NO_3_)_2_·3H_2_O and Zn(CH_3_COO)_2_·2H_2_O, and through hydrothermal synthesis, they obtained ZnO:CuS nanoparticles that exhibited excellent photocatalytic activity. Xu et al. [88] also investigated DES-assisted synthesis for obtaining a photocatalyst. In this case, in the first step, Cd(CH_3_COO)_2_ was solubilized in a DES of EG and thiourea, and spherical CdS was obtained through hydrothermal synthesis. In a second step, these spherical CdS particles were mixed with the same DES, water, and Ce(NO_3_)_3_·5H_2_O, and through a solvothermal treatment followed by a calcination process, the CdS@CeO_2_ photocatalyst composites were obtained.

Other studies, such as that by Jin et al. [89], in which ZnO/graphene nanohybrids were fabricated using a DES based on ChCl and DEG as the reaction medium to dissolve the zinc salt precursor and disperse the graphene, or that by Baby et al. [90], in which AFe_2_O_4_ (A = Mg, Zn or Mn) nanoparticles were synthesized using a DES composed of ChCl and malonic acid as the solvent medium for the precursors α-Fe_2_O_3_ and metal oxides, demonstrate the current potential and viability of DESs as sustainable solvents.

**Table 3:** Different roles of DESs in the synthesis of catalytic materials.

Catalyst	DES	Molar Ratio	Role of DES in Synthesis	References
ZnO/CuS nanoarchitectures	Urea:fructose:water Urea:glucosa:water	1:1:2 1:1:1	Solvent	[87]
CdS@CeO_2_ composites	PEG:thiourea	2:1	Solvent Sulfur source	[88]
ZnO/graphene composites	ChCl:DEG	1:2	Solvent	[89]
AFe_2_O_4_ (A = Mg, Zn, or Mn) nanoparticles	ChCl:malonic acid	1:1	Solvent	[90]
Fe nanoparticles	ChCl:sucrose	2:1	Stabilizer Capping agent	[91]
CeO_2_ nanoparticles	CTAB:acetic acid	1:1	Stabilizer	[92]
ZnO nanoparticles	ChCl:DEG	1:2	Stabilizer	[93]
α-Fe2O3 nanoparticles	ChCl:FeCl3·6H2O	1:2	Solvent Precursor Template	[94]
Zn-doped SnO2 and Zn2SnO4 nanostructures	ChCl:SnCl2 ChCl:ZnCl2	1:2 1:2	Precursor Structure-directing agent	[95]
Fe-hBN nanocomposites	EG:ChCl	2:1	Fe doping	[96]
N-doped biochar	ChCl:Urea	1:2	N doping	[97]
Cl-doped CuO	ChCl:Urea	1:2	Cl doping	[98]
Co-g-C_3_N_4_ Fe-g-C_3_N_4_ CoFe-g-C_3_N_4_	CoCl_2_·6H_2_O:Urea FeCl_3_:Urea CoCl_2_·6H_2_O:FeCl_3_:Urea	1:2 1:1.35 1:1.5:2	Metal doping	[99]
Flower-like hierarchical BiOCl structures	ChCl:Urea	1:2	Solvent Chlorine source Template	[100]
TiO_2_ nanoparticles	ChCl:PTSA	1:1	Template	[101]
TiO_2_ nanoparticles	ChCl:hydroquinone	2:1	Structure-directing agent Capping agent	[102]
Hierarchically nanostructured BiVO_4_	ChCl:Urea	1:2	Structure-directing agent	[103]
Flower-like hierarchical BiOCl/BiVO_4_	ChCl:citric acid	1:1	Structure-directing agent	[104]
Ti_3_C_2_/TiO_2_	ChCl:HPF_6_ ChCl:HBF_4_ ChCl:CF_3_SO_3_H	1:1 1:2 1:3	Intercalation	[105]
Ni-P@POC nanosheets	TBPC:Urea	1:1	Intercalation/etching	[106]

Given that DESs are composed of hydrogen bond interactions, they are usually highly soluble in water. This characteristic directly affects the viscosity of DESs; the addition of water decreases the viscosity of the mixture, facilitating their use as solvents for certain compounds. Some studies, such as that by Negi et al. [87], took advantage of this property and designed a DES/water system. With the aim of obtaining ZnO:CuS nanoparticles, Negi et al. [87] initially attempted to solubilize Cu(NO_3_)_2_·3H_2_O and Zn(CH_3_COO)_2_·2H_2_O in a DES composed of urea, glucose, and water in a 1:1:1 molar ratio, but it turned out to be too viscous and therefore impossible to use as a solvent for the synthesis. By carrying out a series of DES dilutions in water, they managed to reduce the viscosity and finally concluded that, in order to function effectively as a solvent and enable nanoparticle synthesis, the optimal condition was to dilute the DES by adding 100% (*v*/*v*) water compared to the concentrated DES.

The instability of metal compounds is a drawback when working with them in the synthesis of certain materials. Due to their properties, in addition to functioning as solvents, DESs also allow for controlling the stability of metal oxides and are therefore used as stabilizing agents in several studies, especially those related to nanoparticle synthesis. One example is the study conducted by Swathi et al. [91], where a DES based on ChCl and sucrose was used as a stabilizing agent, together with FeCl_3_·6H_2_O and FeCl_2_·4H_2_O as precursors, for the synthesis of Fe nanoparticles. Another example is the method for obtaining CeO_2_ nanoparticles proposed by Iqbal et al. [92]. To synthesize the nanoparticles, they suggested the dropwise addition of a DES based on CTAB and acetic acid into a solution containing ammonium cerium (IV) nitrate, isopropyl alcohol (IPA), and ethylene glycol (EG). Thanks to the stabilizing effect provided by the DES, they prevented particle agglomeration and obtained monodispersed and highly stable CeO_2_ nanoparticles.

Residual DES species may remain adsorbed on the catalyst surface after synthesis, where they can partially block active sites or modify surface properties, such as hydrophilicity, acidity, and surface charge, thereby affecting catalytic activity and reaction pathways. Although in some cases these residues may contribute to improved particle stabilization, their presence cannot be assumed to be inert, particularly in environmental applications. Under advanced oxidation conditions, organic components of DESs may undergo oxidative degradation, potentially generating low-molecular-weight organic by-products or nitrogen- and chlorine-containing species that could act as radical scavengers or introduce undesired secondary contaminants. Therefore, complete removal of DES residues, thorough surface characterization, and appropriate control experiments are essential to ensure reliable evaluation of DES-assisted catalysts and to prevent unintended secondary contamination during catalytic oxidation processes.

#### 3.1.2. DES as Precursor and Doping Component

Beyond being excellent solvents, DESs themselves can act as precursors [80,85]. This is often the case when one of their components contains a metal, as is the case with Type I, II, and IV DESs, which, as explained previously in Table 1, have a metal chloride or a hydrated metal chloride as one of their constituents. Zahmatkeshani et al. [95] were the first to synthesize different hierarchical structures of SnO_2_, Zn-doped SnO_2_, and Zn_2_SnO_4_ via the hydrothermal method, using DESs composed of ChCl with SnCl_2_ and ChCl with ZnCl_2_ as Sn and Zn precursors, without the addition of any other materials. In another study, Karimi et al. [94] used a DES composed of ChCl and FeCl_3_·6H_2_O as an Fe precursor to carry out the synthesis of photocatalytic α-Fe_2_O_3_ nanoparticles.

DESs can also serve as a source of certain elements required for material synthesis. For instance, Xu et al. [88] employed a DES composed of PEG 200 and thiourea in a 2:1 molar ratio to prepare CdS@CeO_2_ composites, where the main function of thiourea was to provide the sulfur needed for the reaction, acting as a source of this element.

**Figure 5:**
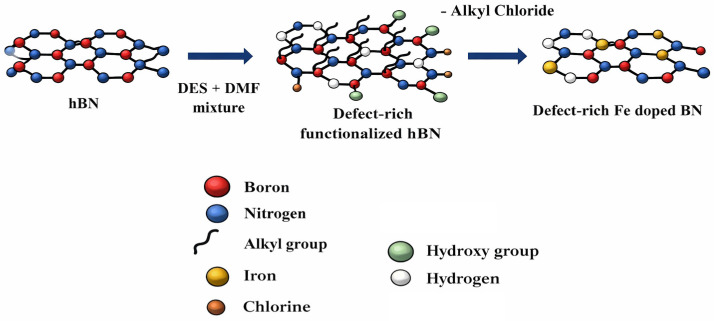
Reaction occurring using DES as a precursor to obtain defect-rich Fe-hBN. Reproduced with permission from ref. [96], Copyright 2025 ACS.

Moreover, due to the ability of DESs to dissolve a wide range of precursors, element-doping can be easily achieved using DES systems. Glass et al. [96] reported a novel DES-based approach to synthesize defect-rich hexagonal boron nitride (hBN) heterogeneously doped with ferric iron, forming Fe-hBN photocatalyst nanocomposites for the removal of perfluorooctanoic acid. In their study, a mixture of FeCl_2_·4H_2_O, hBN, dimethylformamide (DMF), and a DES composed of EG and ChCl in a 2:1 molar ratio was subjected to solvothermal treatment. Heating the DES to 180 °C generated various amine-based products that served as excellent precursors for functionalizing and producing defect-rich hBN, as can be observed in Figure 5. Subsequently, the DES reacted with the iron chloride, displacing alkyl groups and anchoring Fe atoms into the matrix, thus forming the Fe-hBN nanocomposites.

In addition, DESs can also act as a direct source of heteroatoms for the preparation of doped catalysts. For example, Ye et al. [97] synthesized nitrogen-doped biochar using a DES composed of ChCl and urea, where the nitrogen incorporated into the biochar originated from the urea component. Similarly, Jaihindh et al. [98] introduced Cl into the CuO lattice using a DES formed by ChCl and urea. In this system, ChCl provided the Cl- anions, and the doping level was effectively tuned by adjusting the molar ratio of DES components, successfully yielding Cl-doped CuO. Another notable example is the work of Wang et al. [99], where they employed transition metal-based DESs to prepare transition metal-doped carbon nitride catalysts for the photo-Fenton degradation of dyes and antibiotics. In their approach, DESs composed of CoCl_2_·6H_2_O and urea, FeCl_3_ and urea, or a combination of CoCl_2_·6H_2_O, FeCl_3_, and urea were mixed with melamine and cyanuric chloride and subjected to hydrothermal treatment at 180 °C. During this process, the transition metal ions within the DES directly coordinated with the nitrogen atoms of melamine during g-C_3_N_4_ polymerization, leading to efficient metal doping.

#### 3.1.3. DES for Morphology and Structure Control

The surface properties of catalysts play a crucial role, as they directly influence their catalytic performance. For this reason, various strategies have been explored to control or modify the morphology and structure of these materials with the aim of improving their surface characteristics. In this context, several studies have concluded that DESs can effectively function as templates, coating agents, and structure-directing agents [80,85].

**Figure 6:**
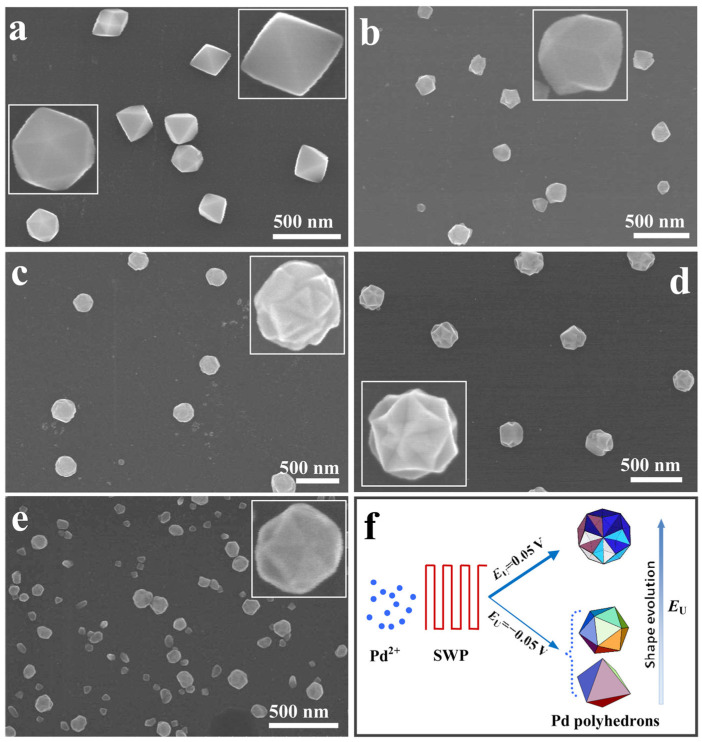
(**a**–**e**) SEM images of Pd NCs electrodeposited on GCE in 1 mM PdCl2-DES solution at 60 °C by square-wave potential: EL = −0.40 V and EU = −0.05, 0, 0.025, 0.05, and 0.10 V, respectively, at f = 10 Hz for 45 min. (**f**) Illustration of shape evolution of polyhedral Pd NCs by adjusting EU. Reproduced with permission from ref. [110], Copyright 2016 ACS.

DESs can organize low-molecular-weight species around them, giving rise to specific geometric topologies; this phenomenon is known as the templating effect. It typically occurs during the nucleation or gelation stages and results from differences in intermolecular interactions, which guide the formation of well-ordered products [80]. The high viscosity of DESs also plays a key role in controlling catalyst morphology by significantly influencing mass transport and solute diffusion. Compared to conventional aqueous solvents, DESs exhibit viscosities one or two orders of magnitude higher at room temperature, which markedly slows the diffusion of metal precursors, ligands, and reactive species [107]. This reduced mass transport can hinder mixing and the uniform delivery of reactants to nucleation sites, thereby slowing the overall nucleation and growth rates [108]. The viscosity of DESs can be reduced by dilution with water, increasing the temperature, or applying forced convection, such as stirring or ultrasound [107]. Importantly, this property can be exploited to achieve morphology control, although it requires careful optimization of reaction conditions to balance mass transport limitations with the desired structural outcomes [109]. Karimi et al. [94] demonstrated the feasibility of using a DES composed of ChCl and FeCl_3_·6H_2_O in a 1:2 molar ratio as a template, successfully synthesizing amorphous α-Fe_2_O_3_ nanoparticles with particle sizes ranging from 25 to 75 nm. Similarly, Sashi et al. [101] employed a DES formed by ChCl and PTSA as a template for the production of TiO_2_ nanoparticles and investigated how variations in DES concentration affect key physicochemical properties of TiO_2_, such as morphology, particle size, and surface area. Wei et al. [110] developed concave-disdyakis triacontahedral Pd nanocrystals via shape-controlled electrochemical synthesis using a ChCl and urea-based DES. The role of the DES and the selection of a proper upper (EU) and lower (EL) limit potential affected the obtained results (Figure 6), remarking the role of the DES in this synthesis.

**Figure 7:**
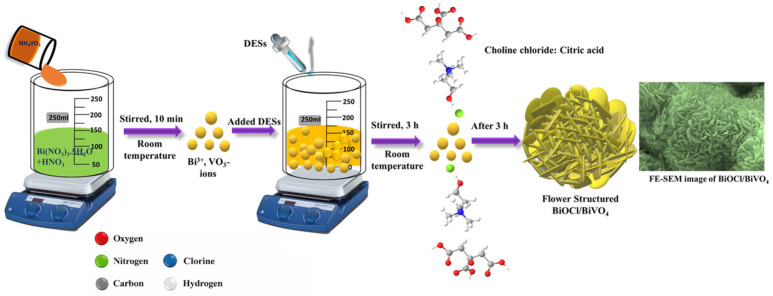
Schematic of the growth mechanism of the hierarchical flower-like BiOCl/BiVO_4_ using a DES as a structure-directing agent. Reproduced with permission from ref. [104], Copyright 2020 Chemistry Europe.

On the other hand, DESs have the ability to direct crystallization toward a specific structural arrangement, a phenomenon known as the structure-directing effect [85]. The components of DES can modulate nucleation and growth mechanisms through charge neutralization, modification of reduction potentials, and passivation of specific crystal facets, thereby guiding growth along preferred crystallographic directions [103]. As a result of the DES’s high viscosity, crystal growth becomes kinetically controlled rather than diffusion-limited, favoring the formation of smaller, more uniform particles and enabling the fine-tuning of morphology, particle size, and surface structure. Sandhu et al. [102] reported a novel synthesis for obtaining TiO_2_ nanoparticles using a DES composed of ChCl and hydroquinone in a 2:1 molar ratio as a structure-directing agent. Likewise, Jaihindh et al. [103] focused their research on the preparation of hierarchically nanostructured BiVO_4_ with a shuriken-like morphology as a bifunctional catalyst for the photocatalytic degradation and electrochemical detection of highly toxic hexavalent chromium. They employed a DES consisting of ChCl and urea as both the reaction medium and shape-controlling agent, which enabled morphology control through one of the least energy-intensive synthesis routes. Elemental mapping images clearly revealed that Bi, V, and O elements were uniformly distributed, confirming the formation of the hierarchical BiVO_4_ structure obtained. In another study, Jaihindh et al. [104] demonstrated a new one-step green synthesis method for the preparation of flower-like hierarchical BiOCl/BiVO_4_ using a DES composed of ChCl and citric acid. In this work, the key factor is the use of the DES as a structure-directing agent. As shown in Figure 7, initially, the mixed solution of Bi(NO_3_)_3_·5H_2_O and NH_4_VO_3_ at room temperature contained Bi^3+^ and VO_3_^−^ ions. Upon addition of the DES to this solution, the bismuth ions preferentially bonded with chloride anions and with the lone pairs of electronegative oxygen atoms. Subsequently, the VO_3_^−^ ions in the solution reacted with the anchored Bi^3+^ ions. Citric acid and choline molecules, as well as water molecules, formed a medium capable of establishing Bi-O bonds while simultaneously generating hydrogen bonding with chloride ions, enabling a more extensive hydrogen bond network within the structure. Importantly, the reactive elements were tightly bound, as bismuth became coordinated by citric acid, which in turn established a strong hydrogen bonding network with water. These reactive bismuth centers, together with the pre-structuring effect of the solvent, effectively reduced the reaction activation energy and promoted the growth of the flower-like structure.

Finally, DESs can be used as capping agents, preventing the growth of nanostructures. With the aim of replacing the polymers and surfactants that typically fulfill this function [91,102], several studies have explored the possibility of using DESs as substitutes, thereby adhering to the principles of green synthesis. Sandhu et al. [102] highlighted the usefulness of the DES composed of ChCl and hydroquinone as a coating agent in the preparation of TiO_2_ nanoparticles. Another example is the work conducted by Swathi et al. [91], in which a DES based on ChCl and sucrose was employed as a coating agent during the synthesis of amorphous Fe nanoparticles. Similarly to those explored in the previous section, under the conditions of AOPs, the organic capping agents, which are constituents of DES, are susceptible to oxidative degradation, potentially yielding products as well as nitrogen- and chlorine-containing species. These transformation products may function as radical scavengers or contribute to the introduction of secondary contaminants, thereby influencing the overall oxidative process.

#### 3.1.4. DES for Intercalation and Exfoliation Processes

Conventional materials that act as catalysts usually possess abundant active sites and a specific surface area. However, spontaneous stacking in some of these materials, due to van der Waals interactions between layers, is inevitable. This reduces their catalytic performance because the number of exposed active sites decreases. DESs can be considered effective intercalation and exfoliation agents, capable of penetrating between the layers of materials to prevent stacking and to obtain single- or few-layer materials with all active sites accessible. This functionality of DESs in the field of catalyst development for AOPs is very recent [80,85].

Song et al. [105] employed DESs in an intercalation strategy with the aim of obtaining a Ti_3_C_2_-Mxene-derived heterojunction photocatalyst with improved properties compared with that obtained through the traditional method. In their study, they observed that the limited interlayer spacing of 2D Ti_3_C_2_ hinders the in situ growth of the TiO_2_ photocatalyst, thereby decreasing its performance. For this reason, they developed an intercalation strategy based on DESs to achieve interlayer expansion of Ti_3_C_2_ and to improve the performance of the Ti_3_C_2_-derived photocatalyst. They prepared three DESs composed of ChCl and HPF_6_, ChCl and HBF_4_, and ChCl and CF_3_SO_3_H, with molar ratios of 1:1, 1:2, and 1:3, respectively. Owing to the intercalation of choline cations, the Ti_3_C_2_ synthesized using DESs exhibited a larger *c*-lattice parameter than the Ti_3_C_2_ obtained via the traditional method. The interlayer space of Ti_3_C_2_ could intercalate a greater amount of water molecules for the oxidation of Ti atoms, which significantly promoted the in situ growth of TiO_2_ crystals. As a result, the Ti_3_C_2_/TiO_2_ photocatalyst showed superior performance in the removal of perfluorooctanoic acid compared with that obtained through the traditional route. Likewise, Xu et al. [106] employed the DES as both etching and source of phosphides in the preparation of a composite with nickel phosphide embedded in carbon films (Ni-P@POC) in order to generate a sheet structure. They used a DES of TBCP and urea with a molar ratio of 1:1, and the generated catalyst exhibited a good performance in its application to the electrooxidation of 5-Hydroxymethylfurfural.

### 3.2. Synthesis of Functionalized Catalyst

After outlining the various roles that DESs can play in catalyst synthesis, this section provides a detailed overview of the most commonly used synthesis methods. Each method that has been most frequently employed in recent years for catalyst synthesis with DESs is discussed and exemplified.

#### 3.2.1. Hydrothermal Synthesis

This synthesis method involves performing a chemical reaction in an aqueous solvent, typically water, under pressure and at a temperature above the solvent’s boiling point within a specialized reactor. The reaction is carried out in a closed vessel, usually made of polypropylene or polytetrafluoroethylene, sealed inside a steel autoclave. This approach offers several advantages, including uniform material dispersion, mild reaction conditions, and enhanced solubility of otherwise insoluble materials due to elevated pressure and temperature without generating harmful by-products. Consequently, hydrothermal synthesis is considered one of the most suitable and widely used conventional techniques for material synthesis [111].

Recently, DESs have been incorporated into hydrothermal processes, particularly for the preparation of photocatalysts, as shown in Table 4. Negi et al. [87] demonstrated that ZnO/CuS nanocomposites with excellent photocatalytic performance for tetracycline and malachite green degradation can be obtained using a NADES composed of urea, glucose, and water through hydrothermal synthesis. Similarly, Barveen et al. [112] employed a DES of ChCl and fructose in combination with the hydrothermal method to synthesize Au-NPS/ZnS nanoflowers.

The integration of DESs into the hydrothermal system can also play an important and significant role in the structural formation of materials during the reaction [111]. Iqbal et al. [92] demonstrated this in their study, in which they performed the hydrothermal synthesis of CeO_2_ nanoparticles using a DES composed of CTAB and acetic acid. By synthesizing the nanoparticles with and without DES, they observed that the CeO_2_ nanoparticles prepared in the presence of DES were less agglomerated and exhibited higher crystallinity and larger surface area compared to those synthesized without DES. Therefore, they confirmed that the morphological structure, crystallinity, and surface area are directly influenced by the DES.

**Figure 8:**
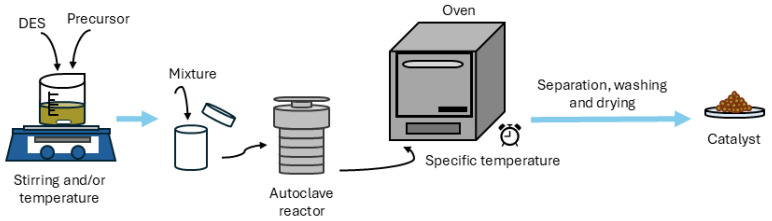
General scheme of the DES-assisted hydrothermal synthesis of a catalyst.

Figure 8 and Table 4 illustrate the typical procedure followed in several studies for the hydrothermal synthesis of catalysts using DESs. As shown, the DES is usually employed in the preparation of the initial mixture, typically as a solvent or precursor. Once the reactants are dissolved, the mixture is transferred to the hydrothermal reactor and placed in an oven where the synthesis occurs at a specific temperature. After the reaction is complete, the system is allowed to cool to room temperature. To obtain the final catalyst, it is usually necessary to perform separation, washing, and drying steps.

#### 3.2.2. Solvothermal Synthesis

The basis and procedure of the solvothermal method are similar to those of the hydrothermal method; the main difference lies in the use of organic solvents as the reaction medium instead of aqueous solutions. This method is characterized by its ability to tailor the properties of the synthesized materials by varying the type of solvent employed, since each solvent, under high pressure and temperature, can exert different effects on the precursors [111,115]. Commonly used organic solvents include acetonitrile, methanol, acetone, and DMF.

A major advantage of the solvothermal method compared to other synthesis techniques is the high crystallinity and the excellent structural and morphological properties of the final materials, which can be achieved by adjusting the reaction parameters. However, the prolonged synthesis time and the use of environmentally unfriendly solvents represent the main limitations of this technique [111,115].

Due to their role as precursors, structure-directing agents, and templates, DESs have recently been incorporated into solvothermal synthesis, as reported in several studies summarized in Table 4. Notably, Glass et al. [96] innovatively employed a DES composed of EG and ChCl together with DMF as precursors in solvothermal synthesis to obtain defect-rich, functionalized, and heterogeneously iron-doped hBN, forming Fe-hBN photocatalytic nanocomposites. In another study, Ge et al. [100] performed the solvothermal synthesis of hierarchical BiOCl structures with flower-like morphology using a DES composed of ChCl and urea. In this case, the DES played a dual role, serving both as a direct source of Cl^−^ ions and as a structure-directing agent to modulate the morphological properties of the catalyst. In the same study, the synthesis was also carried out using alternative chlorine sources such as NaCl, demonstrating that the desired flower-like morphology was achieved only when the DES was used as one of the precursors.

#### 3.2.3. Precipitation Synthesis

**Figure 9:**
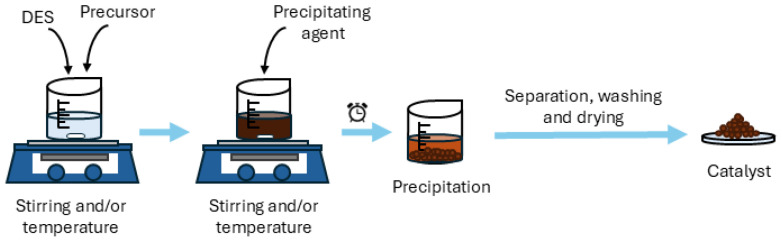
General scheme of the main stages involved in the DES-assisted precipitation synthesis process.

Precipitation is another effective technique for catalyst preparation. In general, as can be seen in Figure 9, this method involves an initial stage in which the precursor compounds are dissolved in a solvent, typically assisted by heating and stirring, followed by the addition of a precipitating agent that leads to the formation of the desired material. Finally, the resulting solid is usually separated by centrifugation or filtration and subsequently washed and dried. Several factors can influence the process and consequently the properties of the final material, including temperature, pH, reaction time, stirring rate, as well as the concentration and nature of the precursors [111].

The incorporation of DES in the initial stage of the synthesis (Table 5) improves the solubilization of the precursors and enables enhanced structural control, particularly in the synthesis of nanoparticles, providing greater stability and precise control over particle size [111]. In this context, Cun et al. [93] employed a DES composed of ChCl and DEG as the solvent to dissolve Zn(CH_3_COO)_2_·2H_2_O, followed by the addition of NaOH as the precipitating agent, obtaining ZnO nanoparticles with excellent photocatalytic activity in the degradation of methylene blue. Similarly, Sakthi et al. [116] reported the synthesis of Fe_3_O_4_ nanocubes using a DES based on ChCl and citric acid. In this case, FeCl_2_·4H_2_O and FeCl_3_·6H_2_O were dissolved in the DES, and precipitation was induced by the addition of KOH. SEM and TEM analyses demonstrated that DES played a crucial role in controlling particle size and determining the morphology of the obtained material. Furthermore, XRD analysis confirmed the formation of pure magnetite phases exhibiting an inverse cubic spinel structure, with an average crystallite size of 6.13 nm.

In some cases, the precipitate obtained after washing is subjected to high-temperature treatments, such as calcination or pyrolysis, to achieve the desired material. This approach was employed by Verma et al. [117], who synthesized CuO/g-C_3_N_4_ composites via DES-assisted chlorine doping. Another example is the study by Jaihindh et al. [104], in which precipitation followed by calcination enabled the DES-assisted synthesis of flower-like BiOCl/BiVO_4_ structures and ternary heterojunctions with g-C_3_N_4_.

#### 3.2.4. Pyrolysis and Calcination Synthesis

Calcination and pyrolysis are both thermal decomposition processes commonly employed in catalyst synthesis. Calcination is carried out in the presence of oxygen at temperatures ranging from 200 °C to 1200 °C, whereas pyrolysis takes place in the absence of oxygen at lower temperatures, typically between 300 °C and 800 °C. In general, the synthesis of catalysts using these methods involves an initial stage in which a given precursor is dissolved in a solvent medium, followed by a second stage in which the resulting mixture is subjected to a thermal treatment (calcination or pyrolysis) at a selected temperature for a defined period of time, leading to the formation of the desired material [115].

This approach is widely applied in the synthesis of catalytic materials for AOPs. As an alternative to conventional solvents, the incorporation of DES during the synthesis process enables a homogeneous distribution of the chemical species, resulting in improved calcination or subsequent pyrolysis.

**Figure 10:**
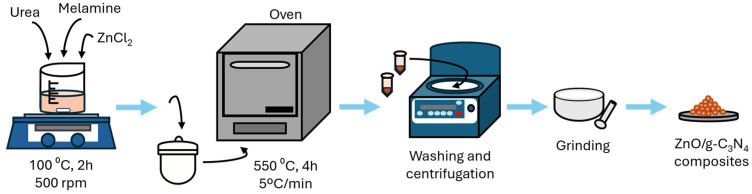
Scheme of the steps carried out in the DES-assisted calcination synthesis for the preparation of ZnO/g-C_3_N_4_ [118].

Recently, Lomba et al. [118] demonstrated that ZnO/g-C_3_N_4_ composites can be synthesized via the calcination of a ternary DES composed of urea, ZnCl_2,_ and melamine, as can be seen in Figure 10. In another study, He et al. [119] successfully prepared iron-doped carbon nanotube catalysts (Fe-CNTs) through DES-assisted pyrolysis of residual biomass. The synthesized catalyst was subsequently immobilized on a glass fiber electrode and employed as a cathode in the electro-Fenton degradation of Rhodamine B, achieving excellent performance. These results demonstrated that this synthesis strategy offers significant advantages in terms of environmental friendliness, pollution-free operation, and high efficiency compared to conventional methods.

#### 3.2.5. Electrochemical Synthesis

This synthesis technique involves the use of electric currents to induce chemical reactions that allow for the formation of catalytic materials directly on an electrode or within an electrolyte. Normally, this process takes place in an electrochemical cell, which requires a power supply, an anode, and a cathode. Metallic precursors or compounds of interest are dissolved in a conductive medium (electrolyte) inside the cell, where the electrodes are immersed. Then, using the power supply, a controlled voltage is applied to generate a potential difference between the electrodes, causing the metal ions to be reduced or chemically combined on the electrode surface or within the electrochemical medium itself, forming the desired catalyst [111].

DESs have emerged as promising electrolytes for electrosynthesis due to their high ionic conductivity, strong solvation ability, and wide electrochemical window [111,115]. Several studies have investigated the efficiency of DES-assisted electrosynthesis. Notably, Jia et al. [120] proposed and implemented an electrolysis strategy using a DES for the preparation of N and P-co-doped TiO_2_. In their study, the electrolyte consisted of a DES formed by ChP and urea mixed with dimethyl sulfoxide, and high-purity titanium plates served as the cathode and the anode. Upon application of the electric current, titanium oxidation occurred, which reacted with the active oxygen generated from the DES electrolysis to form titanium oxide. Furthermore, N- and/or P-containing species resulting from DES decomposition adhered to the titanium oxide, yielding N and P-co-doped TiO_2_.

#### 3.2.6. Other Methods

In addition to the synthesis methods previously described, there are other less commonly used techniques, such as ionothermal, ultrasound-assisted, and sol–gel synthesis.

The ionothermal method is characterized by the absence of solvents in the reaction medium. It is similar to hydrothermal and solvothermal methods; however, in this case, the synthesis occurs in an ionic medium that acts simultaneously as a solvent and a structure-directing agent or template. The availability of DESs formed from high-boiling neutral compounds, such as urea, carboxylic acids, or carbohydrates, provides an opportunity for the fabrication of new materials using this technique. Ciğeroğlu et al. [121] employed this method in their study to achieve the green and one-step preparation of a Zn/GO nanocomposite assisted by a DES composed of ChCl and EG.

Regarding ultrasound-assisted catalyst synthesis, it is based on cyclic mechanical vibrations at a specific frequency that facilitate the chemical reaction. Its main advantage is its high energy efficiency, as the process occurs under ambient conditions. Few studies have combined this method with DESs. However, recently, Negi et al. [122] prepared a series of g-C_3_N_4_ photocatalysts using different NADES through this approach, aiming to controllably modify g-C_3_N_4_ and enhance its performance.

## 4. Application in AOPs

AOPs are based on the generation of highly reactive oxidizing species (ROS), such as hydroxyl (•OH), sulfate radicals (SO_4_•^−^), hydroperoxyl (HO_2_•), superoxide (O_2_•^−^), and peroxyl (RO_2_•), and non-radicals, such as singlet oxygen (^1^O_2_) and hydrogen peroxide (H_2_O_2_). These ROS oxidize and degrade contaminants until they are partially mineralized, resulting in intermediate products, or completely mineralized, yielding CO_2_, H_2_O, and inorganic compounds. The versatility of these processes lies in the fact that there are different ways to produce these radicals, allowing for the selection of the most suitable method depending on the specific requirements. AOPs have emerged as powerful techniques for the degradation of persistent organic pollutants due to their high oxidation capacity, wide applicability, and fast reaction rates. Therefore, this section will provide a comprehensive overview of the application of DES-assisted synthesized catalysts in the most common AOPs for water treatment, including photocatalysis, Fenton, photo-Fenton, and electro-Fenton.

### 4.1. Photocatalysis

In this AOP, oxidant radicals are generated through radiation from a light source, which can be artificial, such as ultraviolet (UV) light or visible light, or natural, such as sunlight. In this process, photocatalysts are typically used to promote the reaction and generate reactive species, such as superoxide (O_2_•^−^), hydroxyl (•OH), holes (h^+^), electrons (e^−^), and singlet oxygen (^1^O_2_). These photocatalysts are semiconductor materials and are defined by having two distinct energy bands. A higher energy band that contains electrons, known as the conduction band, and a lower energy band without electrons, known as the valence band. Traditionally, TiO_2_ is the most studied and well-known photocatalyst due to its high stability, low toxicity, and low cost. In recent years, graphitic carbon nitride (g-C_3_N_4_) has emerged as a greener alternative, and its applications in photocatalysis have been extensively explored. However, recent advances in DES-assisted synthesis have led to the development of other photocatalysts, and modifications to both TiO_2_ and g-C_3_N_4_ have been made to improve their photocatalytic activity.

Table 6 below summarizes the most recent publications related to photocatalysts synthesized via DES. It can be observed that the degradation efficiency of pollutants generally exceeds 90%, demonstrating the excellent photocatalytic capacity of these materials. This also highlights that the various roles played by DES during synthesis, as previously explained, facilitate photocatalytic enhancement strategies, such as junction formation, facet exposure, surface defects, and crystalline phase composition. Additionally, several studies show that the use of DESs as a synthesis medium enables the tuning of the photoactive response range through doping and the combination with carbon-based materials.

Iqbal et al. [92] observed that the CeO_2_ nanoparticles synthesized using DES as a solvent had a larger surface area compared to the same nanoparticles synthesized with other reaction media. The superior Flumequine removal efficiency of the nanoparticles synthesized with DES (94%) compared to those without DES (73%) demonstrated that the mere participation of DES as a solvent in the synthesis provided the material with an optimal surface area, which favored the photocatalytic behavior by facilitating the access of target molecules to the catalyst’s active sites. Furthermore, in the same study, the role of DES as a stability enhancement agent was confirmed. Reusability cycles of the CeO_2_ nanoparticles synthesized via DES showed 68% degradation after six cycles, while nanoparticles synthesized using the conventional method showed only 25% degradation due to the proposed degradation pathway under UV irradiation without a catalyst.

In other studies of interest, DES was used to modify g-C_3_N_4_, increasing its photocatalytic performance. For example, during the DES-assisted synthesis of Cl-CuO/g-C_3_N_4_, Cl^−^ anions from DES were introduced into the oxygen sites of the metal oxides, achieving a degradation of 92.8% of 4-Nitrophenol. This high photocatalytic activity was associated with the presence of a large number of reactive sites on the catalyst’s surface, enabling rapid charge transport and also reducing the electron-hole recombination rate after photoexcitation [98]. Negi et al. [122] also based their research on modifying g-C_3_N_4_ to achieve greater photocatalytic performance. Their intention was to use NADES with different pH values for the first time as self-assembly control agents for g-C_3_N_4_. They designed different NADES with neutral, acidic, and basic pH, and for proper comparison with conventional exfoliation solvents, g-C_3_N_4_ was also modified in DW and IPA. The g-C_3_N_4_ modified with an acidic NADES exhibited the most exfoliated topology and the largest surface area, a significant increase in the magnitude of the negative surface charge potential compared to unmodified g-C_3_N_4_, and an increase in the band gap. Additionally, the g-C_3_N_4_ modified with an acidic NADES showed the highest degradation efficiency, confirming that the modification of g-C_3_N_4_ using NADESs, particularly acidic NADESs, was an excellent strategy for improving the photocatalytic properties of g-C_3_N_4_.

### 4.2. Fenton and Photo-Fenton

The Fenton process is based on the generation of •OH by the H_2_O_2_ decomposition in the presence of metal salts, typically iron salts, in solution. As shown in Equations (2)–(7), the externally added H_2_O_2_ is reduced, leading to the formation of •OH radicals due to the oxidation of iron, which changes from Fe^2+^ to Fe^3+^ (Equation (2)). The generated radicals react with contaminants (RH), degrading them (Equation (3)). Additionally, the •OH radicals continue to react with the remaining Fe^2+^ in the solution until it is completely converted to Fe^3+^ (Equation (4)). Finally, the Fe^3+^ ion can be reduced by reacting with H_2_O_2,_ forming the Fe^2+^ ion again and lower amounts of hydroperoxyl (HO_2_•) (Equations (5)–(7)). This process is slower but allows for the regeneration of Fe^2+^.

(2) $${Fe}^{2+}+H_{2}O_{2}\to {Fe}^{3+}+•OH+{OH}^{-}$$

(3) $$RH+•OH\to R•+H_{2}O$$

(4) $${Fe}^{2+}+•OH\to {Fe}^{3+}+{OH}^{-}$$

(5) $${Fe}^{3+}+H_{2}O_{2}\to {FeOOH}^{2+}+H^{+}$$

(6) $${FeOOH}^{2+}\to {HO}_{2}•+{Fe}^{2+}$$

(7) $${Fe}^{3+}+{HO}_{2}•\to {Fe}^{2+}+H^{+}+O_{2}$$

Although some studies are based on the combination of this process with catalysts synthesized via DES [125,126], as shown in Table 7, yielding good results in the removal of contaminants, most of them use Fenton in combination with photocatalysis or electrocatalysis, resulting in photo-Fenton and electro-Fenton processes.

The photo-Fenton process is a combination of photocatalysis and the Fenton reaction. As shown in Table 7, several studies used DES-based catalysts to carry out the degradation of contaminants in water via this technique, obtaining promising results. One example is the study conducted by Lomba et al. [118], where they used ZnO/g-C_3_N_4_ nanocomposites obtained through the calcination of a DES as catalysts for the photo-Fenton process in the degradation of Rhodamine B. Similarly, Wang et al. [99] and Sakthi et al. [116] carried out the degradation of the same dye but using other catalysts, CoFe-CN composites and Fe_3_O_4_ nanocubes, respectively. In all three studies, the photocatalytic activity of the materials was improved with the addition of H_2_O_2_ to the system.

### 4.3. Electro-Fenton

The electro-Fenton process is based on the in situ electrochemical generation of ∙OH through the application of an electric field. To carry out the process, an electrochemical cell is required, within which the electrolyte is contained, along with a power supply, a cathode, an anode, an air diffuser that supplies oxygen to the system, and a catalyst, which is usually metallic. Typically, H_2_O_2_ is continuously produced at the cathode through the electrochemical reduction of dissolved oxygen supplied by the air diffuser (Equation (8)), while Fe^2+^ acts as a catalyst in the Fenton reaction, reacting with H_2_O_2_ to generate •OH radicals (Equation (2)). These •OH radicals then react with the contaminant, resulting in degradation products and H_2_O (Equation (3)). The electrochemical regeneration of Fe^2+^ from Fe^3+^ allows for the efficient maintenance of the process (Equation (9)).

(8) $$O_{2}+{2H}^{+}+{2e}^{-}\to H_{2}O_{2}$$

(9) $${Fe}^{3+}+e^{-}\to {Fe}^{2+}$$

Therefore, the catalyst and the cathode used are crucial for the process, as they affect the reaction rate and the system’s efficiency. Recently, DES-assisted synthesized catalysts have been implemented in this technique, acting as electrocatalysts by being fixed on the cathode or as heterogeneous catalysts present in the solution. Although there are currently only a few studies available, the degradation results of contaminants, shown in Table 7, are promising.

Highlighting the role of the material as a heterogeneous catalyst in the medium, Lomba et al. [118] synthesized ZnO/g-C_3_N_4_ nanocomposites by calcining a DES and demonstrated their potential as a catalyst in the degradation of Rhodamine B. Using a catalyst dose of 50 mg and applying a current intensity of 25 mA, they achieved almost total degradation of Rhodamine B in 90 min. In another study, Puga et al. [127] synthesized FeTi nanoparticles through DES and achieved total degradation of Lissamine green B and antipyrine by applying the electro-Fenton process. Regarding the fixation of the catalyst on the cathode, in the study by He et al. [119], a Fe-CNT catalyst was prepared through DES-assisted synthesis and then fixed on a graphite-felt cathode. After 5 min of treatment, Rhodamine B was totally degraded, demonstrating the good performance of the modified cathode in the electro-Fenton process.

## 5. Future Perspectives and Conclusions

This review has highlighted the growing synergy between DESs and AOPs, emphasizing their combined potential to contribute to more sustainable water treatment strategies. The versatility of DESs, derived from their adjustable composition and physicochemical properties, allows for their multifunctional role during the synthesis of catalysts. As discussed throughout this review, DESs can act not only as solvents, but also as precursors, dopants, stabilizing agents, templates, structure-directing agents, and intercalation media. This multifunctionality allows for precise control over the catalyst composition, morphology, surface properties, and defect chemistry, which are key parameters governing the catalytic activity in AOPs. Consequently, DES-assisted synthesis routes have allowed for the development of highly efficient catalysts for photocatalysis, Fenton, photo-Fenton, and electro-Fenton processes, often achieving contaminant degradation efficiencies of over 90% under mild operating conditions.

From an environmental and sustainability perspective, the substitution of volatile and toxic organic solvents by DESs represents a significant advance in ecological chemistry. Most DESs are characterized by low volatility, low toxicity, biodegradability, and ease of preparation, often requiring a simple mixture of inexpensive components without purification steps. These characteristics align well with the principles of sustainable chemistry and the circular economy, particularly when DESs are combined with renewable or bio-based components, such as NADES. In addition, the integration of DESs into synthesis pathways has often led to improved catalyst stability and reuse, which are essential factors for real-world applications.

Despite these promising advances, several challenges remain that need to be addressed in future research. One of the main limitations concerns the incomplete understanding of the interactions at the molecular level between the components of the DES, the metal precursors, and the growing nanostructures during the synthesis. A deeper mechanistic view of these interactions would allow for a more rational design of DES formulations adapted to specific catalytic functions. Moreover, although laboratory-scale studies have demonstrated excellent performance, the scalability of DES-assisted synthesis methods remains relatively unexplored. The development of continuous and industrially viable processes, such as twin-screw extrusion or electrochemical routes, will be crucial to move these materials from laboratory research to practical applications.

Another important aspect for future research is the environmental footprint of DESs throughout their entire life cycle. Although DESs are generally considered ecological solvents, systematic life cycle assessments and toxicity studies are still limited. Assessing the destination, recyclability, and potential environmental impacts of DESs after use will be essential to fully validate their sustainability credentials. A particular challenge identified in this context is the recycling of DESs and their related energy/cost requirements. Their composition can change during use due to water absorption, component loss, or chemical degradation, and such changes can alter their physicochemical properties, affecting viscosity, solubilizing ability, and the reproducibility and efficiency of catalyst synthesis. To overcome these limitations, strategies such as compositional adjustment of recycled DESs, careful drying to remove absorbed water, selection of chemically stable formulations, and the design of closed-loop or continuous processes have been proposed. Emerging approaches also explore self-regenerating DES systems capable of maintaining their properties over multiple cycles. Addressing these issues will be critical not only for the sustainable use of DESs themselves but also to ensure that DES-derived catalysts can perform reliably in real-world applications, such as complex wastewater matrices, rather than idealized model solutions.

In conclusion, DESs have proved to be a highly promising ecological means for the synthesis of advanced catalytic materials applied in AOPs for water treatment. Their unique ability to combine sustainability with greater control over catalyst properties positions them as key enablers in the development of next-generation water treatment technologies. Ongoing interdisciplinary research integrating chemistry, materials science, and environmental engineering is expected to further expand the scope of DES-assisted catalyst synthesis, paving the way to more efficient, environmentally friendly, and regulation-compliant wastewater treatment solutions.

## Figures and Tables

**Table 1:** Main types of DES according to their composition.

Type	Formation	Formula	Example	References
I	Quaternary ammonium salt + anhydrous metal chloride	Cat^+^X^−^ + zMCl_x_ M = Zn, Sn, Al, Ga, Fe, In	ChCl + ZnCl_2_	[44]
II	Quaternary ammonium salt + metal chloride hydrate	Cat^+^X^−^ + zMCl_x_ · yH_2_O M = Co, Cu, Ni, Fe, Cr	ChCl + CoCl_2_·6H_2_O	[44]
III	Quaternary ammonium salt + HBD	Cat^+^X^−^ + zRZ Z = OH, COOH, CONH_2_	ChCl + urea	[36]
IV	Metal chloride hydrate + HBD	MCl_x_ + RZ M = Zn, Al Z = OH, CONH_2_	ZnCl_2_ + urea	[36]
V	Non-ionic HBA + HBD	R′Z′ + RZ Z and Z′ = OH, COOH, CONH_2_	Citric acid + sucrose	[43]

**Table 2:** Advantages, disadvantages, and examples of different DES preparation methods.

Method	Advantages	Disadvantages	References
Conventional heating and stirring	- Simple - Fast - Solvent-free - Minimal equipment requirements - Cost-effective	- High energy consumption - Possible degradation of heat-sensitive components	[55,56,57]
Grinding	- Solvent-free - No heating - Environmentally friendly	- Long processing times - Low yields - Not suitable for liquid HBDs	[55,58]
Microwave-assisted preparation	- Fast - Energy-efficient - Cheaper	- Possible degradation of heat-sensitive components	[56,59,60]
Ultrasonic-assisted preparation	- Fast mixing - Mild conditions	- Low energy efficiency	[56,59,61,62,63]
Freeze-drying	- High DES purity - Low risk of thermal degradation	- High energy consumption - High operational cost - Long processing times - Labor-intensive	[52,64,65]
Vacuum-drying	- Removal of volatile impurities - Effective for high viscosity DESs - Improves mixing of solid components - Allows for control of final water content - Good reproducibility	- Requires specialized and expensive equipment - High energy consumption - Risk of composition changes during evaporation	[66]

**Table 4:** Examples of catalysts that are obtained by hydrothermal and solvothermal syntheses.

Catalyst	Method	DES Preparation	Catalyst Synthesis	Reference
CeO_2_ nanoparticles	Hydrothermal	CTAB:acetic acid 1:1 molar ratio Stirred at 70 °C for 3 h	In total, 10 mL of IPA, 10 mL of EG, and 0.3 g of NH_4_·Ce(MO_3_)_6_ were mixed. To this solution, 1 mL of DES was added dropwise under vigorous stirring, and the mixture was stirred for 2 h. The resulting solution was transferred to an autoclave at 130 °C for 7 h. The resulting nanoparticles were separated by centrifugation, washed with EtOH and AC, and dried.	[92]
N-doping CeO_2_ nanoparticles	Hydrothermal	CTAB:acetic acid 1:1 molar ratio Stirred at 70 °C for 3 h	In total, 1 g of NH_4_·Ce(MO_3_)_6_ and 10 mL of water were mixed with another solution consisting of 10 g of urea and 30 mL of water. The mixture was left to rest for 30 min, then 0.5 g of DES was added under stirring. The resulting solution was transferred to an autoclave at 130 °C for 7 h. The resulting nanoparticles were separated by centrifugation, washed with EtOH and AC, and dried.	[113]
TiO_2_ nanoparticles	Hydrothermal and calcination	ChCl:hydroquinone 2:1 molar ratio Stirred at 80 °C	In total, 3 mL of DES was mixed with 1 mL of TBT and 5 mL of DW and stirred at room temperature for 30 min. The mixture was transferred to an autoclave at 150 °C for 5 h. The resulting product was separated by centrifugation, washed with DW and EtOH, and dried overnight under vacuum at 70 °C. The product was calcined at four different temperatures: 100, 350, 550, and 750 °C for 3 h at a heating rate of 3 °C·min^−1^.	[102]
Zn doped SnO_2_ and Zn_2_SnO_4_ nanostructures	Hydrothermal	ChCl:SnCl_2_ ChCl:ZnCl_2_ Both 1:2 molar ratio Stirred at 100 °C for 1 h	Both DESs were mixed with 25 mL of DW. The pH of the mixture was adjusted with HCl and NaOH in the range of 1 to 13. The resulting solution was transferred to an autoclave and subjected to hydrothermal treatment at temperatures ranging from 120 to 220 °C for 24 h. The obtained powders were washed several times with DW and EtOH and dried in air at 60 °C for 12 h.	[95]
Hierarchically nanostructure BiVO_4_	Hydrothermal and calcination	ChCl:urea 1:2 molar ratio Stirred at 80 °C for 30 min	In total, 2.425 g of Bi(NO_3_)_3_·5H_2_O was dissolved in 30 mL of DW, then 0.2 mL of HNO_3_ was added to adjust the pH to 1. The solvent mixture containing 0.584 g of NH_4_VO_3_ was added and stirred for 15 min. DES was added, and the mixture was subjected to hydrothermal treatment at 200 °C for 5, 10, and 15 h. The precipitate was separated, washed with DW and EtOH, and dried overnight at 75 °C. The obtained product was calcinated at 500 °C for 2 h.	[103]
Au-NPS/ZnS nanoflowers	Hydrothermal	ChCl:fructose 1:2 molar ratio	A 0.05 M solution of Zn(CH_3_COO)_2_·2H_2_O and a 0.05 M solution of Na_2_S·9H_2_O were ground in a stoichiometric 1:1 ratio. The mixture was then sonicated in 50 mL of DES. The resulting solution was transferred to an autoclave at 180 °C for 12 h. The obtained powder was separated by centrifugation and washed with EtOH and DW.	[112]
ZnO/CuS nanocomposites	Hydrothermal	Urea:glucosa:water 1:1:1 molar ratio Stirred at 60 °C	Solution A (14.4 g of NaOH, 80 mL of DES, 6.435 g of Zn(CH_3_COO)_2_·2H_2_O, and polyethylene glycol) was mixed with Solution B (2.145 g of Cu(NO_3_)_2_·3H_2_O, 1.522 g of thiourea, and 90 mL of DES). The resulting mixture was transferred to an autoclave and maintained at 120 °C for 24 h. The nanoparticles were washed with DW and EtOH and dried.	[87]
Cu_2_S@MoS_2_ nanoparticles	Hydrothermal	ZnCl_2_:urea 1:3.5 molar ratio Stirred at 100 °C for 90 min	H_24_Mo_7_N_6_O_24_·4H_2_O, Cu(CH_3_COO)_2_·H_2_O, and thioacetamide in a molar ratio of 1:1:2 were dissolved in varying proportions of DES and water. The mixture was transferred to an autoclave and heated at 180 °C for 24 h. The resulting nanoparticles were collected, washed several times with DIW, and dried at 60 °C for 12 h.	[114]
Fe-hBN nanocomposites	Solvothermal	EG:ChCl 2:1 molar ratio	In total, 39.76 mg of FeCl_2_·4H_2_O was added to 496 mg of hBN. To dry the mixture, a solution of 3 mL of DMF and 5 mL of DES was added and stirred. The resulting mixture was transferred to an autoclave at 180 °C for 18 h. The obtained samples were washed with an HEX/EtOH mixture (1:1 *v*/*v*) and dried overnight.	[96]
Flower-like hierarchical BiOCl structures	Solvothermal	ChCl:urea 1:2 molar ratio	In total, 0.001 mol of Bi(NO_3_)_3_·5H_2_O was dissolved in 20 mL of ethylene glycol, then 10 mmol of DES was added and stirred for 30 min. The resulting mixture was transferred to an autoclave and heated at 140 °C for 24 h. The particles were separated, washed with DW and EtOH, and dried under vacuum at 50 °C for 24 h.	[100]

**Table 5:** Examples of catalysts that are obtained by precipitation, calcination, pyrolysis, or electrochemical synthesis.

Catalyst	Synthesis	DES Preparation	Catalyst Synthesis	Reference
ZnO nanoparticles	Precipitation	ChCl:DEG 1:2 molar ratio Stirred at 80 °C	In total, 2.19 g of Zn(CH_3_COO)_2_·2H_2_O was dissolved in 30 mL of DES under magnetic stirring, then 0.8 g of NaOH was added. The mixture was heated under reflux at 120 °C for 6 h. The resulting precipitate was washed several times with DW, EtOH, and AC, and dried under vacuum at 60 °C.	[93]
Fe_3_O_4_ nanocubes	Precipitation	ChCl:citric acid 2:1 molar ratio Stirred at 80 °C for 2 h	Together, 3.9813 g of FeCl_2_·4H_2_O and 8.1091 g of FeCl_3_·6H_2_O were mixed with DES at 80 °C for 20 min under stirring, then 40 mg of KOH was added, and the mixture was stirred for 1 h. The resulting nanoparticles were washed several times with EtOH and DW and dried in a hot air oven.	[116]
Cl-CuO/g-C_3_N_4_	Precipitation and calcination	ChCl:urea 1:2 molar ratio Stirred at 80 °C for 30 min	In total, 1.125 g of Cu(NO)_3_·3H_2_O was dissolved in 60 mL of water and mixed with DES. NaOH was then added to adjust the pH to 10. The mixture was stirred at room temperature for 2 h. The resulting precipitate was washed several times with distilled DW and EtOH and dried at 75 °C overnight. The obtained powder was calcinated at 400 °C for 2 h with a heating rate of 5 °C·min^−1^.	[117]
Flower-like hierarchical BiOCl/BiVO_4_	Precipitation and calcination	ChCl:citric acid 1:1 molar ratio Stirred at 80 °C for 30 min	In total, 2.425 g of Bi(NO_3_)_3_·5H_2_O was dissolved in 50 mL of distilled water, then 0.2 mL of HNO_3_ was added to adjust the pH to 1. The solvent mixture containing 0.584 g of NH_4_VO_3_ was added and stirred for 15 min. DES was then added and stirred at room temperature for 3 h. The resulting precipitate was washed with DW and EtOH and dried overnight at 75 °C. The obtained sampled were calcined at 500 °C for 2 h.	[104]
ZnO/g-C_3_N_4_ composites	Calcination	Urea:ZnCl_2_:melamine 24:1:2 molar ratio Stirred at 100 °C for 2 h	DES was heated to 550 °C for 4 h with a heating rate of 5 °C·min^−1^. Then, it was washed several times with DW and EtOH and dried under vacuum overnight at 60 °C.	[118]
Fe-CNTs	Pyrolysis	Oxalic acid:ChCl:EG 1:1:1 molar ratio Stirred at 120 °C for 2 h	The walnut powder was mixed with the DES (1:15 mass ratio) and stirred at 120 °C for 4 h. The mixture was then combined with EtOH(1:15 volume ratio), filtered, and washed with DW until a neutral pH was reached. The resulting material was mixed with silicon carbide and pyrolyzed at 800 °C for 1 h with a heating rate of 50 °C·min^−1^.	[119]
N and P- co-doped TiO_2_	Electrochemical	ChP:urea 1:2 molar ratio Stirred at 100 °C	DES was diluted with DMSO, and this mixture was used as an electrolyte. Titanium plates were employed as the cathode and the anode. A current density of 40 mA·cm^−2^ was applied. Upon completion of the electrolysis, the insoluble dispersions present in the electrolyte were collected and alternately washed with DW and EtOH.	[120]

**Table 6:** Degradation achieved by the photocatalyst prepared through DES-assisted synthesis methods.

Catalyst	DES (Molar Ratio)	Light Source	Targeted Pollutant	C_o_ (mg/L)	Degradation Efficiency (%)	Time (h)	Reference
CeO_2_ nanoparticles	CTAB:acetic acid (1:1)	UV-C	Flumequine	10	94.00	2	[92]
N-doping CeO_2_ nanoparticles	CTAB:acetic acid (1:1)	Solar	Sulfamethoxazol	10	96.00	2.5	[113]
TiO_2_ nanoparticles	ChCl:hydroquinone (2:1)	UV	Methyl orange	20	-	-	[102]
Hierarchically nanostructured BiVO_4_	ChCl:urea (1:2)	UV	K_2_Cr_2_O_7_	100	95.09	2.67	[103]
Hierarchical flower-like Zn-doped SnO_2_	ChCl:SnCl_2_ (1:2) ChCl:ZnCl_2_ (1:2)	UV	Methyl orange	10	99.50	0.25	[95]
Ti_3_C_2_/TiO_2_	ChCl:HPF_6_ (1:2)	UV	Perfluorooctanoic acid	-	100	16	[105]
CdS@CeO_2_	PEG:thiourea	Visible	Tetracycline	-	91.50	1	[88]
Flower-like BiOCl	ChCl:urea	Sunlight	Rhodamine B	10	98.50	1.25	[100]
ZnO/GO nanocomposite	ChCl:EG (1:2)	UV-A	Cefixime trihydrate	20	86.00	-	[121]
ZnO nanoparticles	ChCl:DEG (1:2)	UV-A	Methylene blue	20	-	-	[93]
3D porous g-C_3_N_4_ nanosheets@carbonized kapok fiber composites	ChCl:glucose (2:1)	Visible	Rhodamine B	10	96.90	3.33	[123]
Cu_2_S@MoS_2_ nanoparticles	ZnCl_2_:urea (1:3.5)	Visible	Rhodamine B Tetracycline	20 25	96.00 97.00	1.5	[114]
Au-NPs/ZnS NFs	ChCl:fructose	UV-C	Toluidine blue	-	97.50	2.5	[112]
Fe-hBN nanocomposites	EG:ChCl (2:1)	UV-C	Perfluorooctanoic Acid	50	-	-	[96]
ZnO/CuS nanoarchitectures	Urea:fructose:water (1:1:2) Urea:glucose:water (1:1:1)	Visible	Tetracycline hydrochloride Malachite green	15 15	99.00 97.00	2	[87]
A-NADES g-C_3_N_4_	Citric acid:fructose:water (1:1:2) Sucrose:fructose:water (1:1:6) Urea:fructose:water (2:1:2)	Visible	Methylene blue Ciprofloxacin	10 15	100 97.60	1	[122]
LDH-Cu_2_O-NADES nanocomposites	Betaine:Glucose:PD	UV-A Sun light	Trypan blue	200	92.80 92.60	1	[124]

**Table 7:** Degradation achieved in Fenton, photo-Fenton, and electro-Fenton processes by the prepared through DES-assisted synthesis methods.

Application	Catalyst	DES (Molar Ratio)	Targeted Pollutant	C_o_ (mg/L)	Degradation Efficiency (%)	Time (min)	Reference
Fenton	Magnetic PolyHIPE nanocomposites	Menthol:acetic acid (1:1)	Gentian violet Methyl orange Neutral red	20 20 20	98.50 98.20 97.10	480	[125]
Fenton	Fe-doped porous activated carbons	ChCl:FeCl_3_	Methylene blue	100	99.00	30	[126]
Photo-Fenton	ZnO/g-C_3_N_4_ nanocomposites	Urea:ZnCl_2_:melamine (24:1:2)	Rhodamine B	10	95.00	60	[118]
Photo-Fenton	Fe_2_O_3_ nanocubes	ChCl:citric acid (2:1)	Rhodamine B	10	94.04	180	[116]
Photo-Fenton	CoFe-CN composites	CoCl_2_·6H_2_O:FeCl_3_:urea	Rhodamine B	5	100	6	[99]
Electro-Fenton	ZnO/g-C_3_N_4_ nanocomposites	Urea:ZnCl_2_:melamine (24:1:2)	Rhodamine B	10	99.21	90	[118]
Electro-Fenton	FeTi nanoparticles	ChCl:urea (2:1)	Lissamine green B Antipyrine	25 25	100 100	80 90	[127]
Electro-Fenton	Fe-CNTs	Oxalic acid:ChCl:EG (1:1:1)	Rhodamine B	50	100	5	[119]

## Data Availability

No new data were created or analyzed in this study. Data sharing is not applicable to this article.

## Abbreviations

AC	Acetone
AADES	Amino acid deep eutectic solvent
AOPs	Advanced oxidation processes
CF_3_SO_3_H	Trifluoromethanesulfonic acid
ChCl	Choline chloride
ChP	Choline phosphate
CTAB	Cetyltrimethylammonium bromide
DEG	Diethylene glycol
DES	Deep eutectic solvent
DIW	Deionized water
DMF	N,N-dimethylformamide
DMSO	Dimethyl sulfoxide
DW	Distilled water
EG	Ethylene glycol
EtOH	Ethanol
HBA	Hydrogen bond acceptor
HBD	Hydrogen bond donor
HBF_4_	Tetrafluoroboric acid
hBN	Hexagonal boron nitride
HEX	Hexane
HPF_6_	Hexafluorophosphoric acid
IPA	Isopropyl alcohol
NADES	Natural deep eutectic solvent
PD	Propanediol
PDES	Polymeric deep eutectic solvent
p.e.	Population equivalent
PEG	Polyethylene glycol
ROS	Reactive oxidizing species
SUPRADES	Supramolecular deep eutectic solvent
TBPC	Tetrabutylphosphonium chloride
THEDES	Therapeutic deep eutectic solvent
TNT	Tetrabutyl titanate
XRD	X-ray diffraction
